# A Dominated Coupling From The Past algorithm for the stochastic simulation of networks of biochemical reactions

**DOI:** 10.1186/1752-0509-2-42

**Published:** 2008-05-08

**Authors:** Martin Hemberg, Mauricio Barahona

**Affiliations:** 1 Department of Bioengineering, Imperial College London, South Kensington Campus, London SW7 2AZ, UK; 2 Institute for Mathematical Sciences, Imperial College London, South Kensington Campus, London SW7 2AZ, UK

## Abstract

**Background:**

In recent years, stochastic descriptions of biochemical reactions based on the Master Equation    (ME) have become widespread. These are especially relevant for models involving gene regulation. Gillespie’s    Stochastic Simulation Algorithm (SSA) is the most widely used method for the numerical evaluation of these    models. The SSA produces exact samples from the distribution of the ME for finite times. However, if the    stationary distribution is of interest, the SSA provides no information about convergence or how long the    algorithm needs to be run to sample from the stationary distribution with given accuracy.

**Results:**

We present a proof and numerical characterization of a Perfect Sampling algorithm for the ME of    networks of biochemical reactions prevalent in gene regulation and enzymatic catalysis. Our algorithm combines    the SSA with Dominated Coupling From The Past (DCFTP) techniques to provide guaranteed sampling from    the stationary distribution. The resulting DCFTP-SSA is applicable to networks of reactions with uni-molecular    stoichiometries and sub-linear, (anti-) monotone propensity functions. We showcase its applicability studying    steady-state properties of stochastic regulatory networks of relevance in synthetic and systems biology.

**Conclusion:**

The DCFTP-SSA provides an extension to Gillespie’s SSA with guaranteed sampling from the    stationary solution of the ME for a broad class of stochastic biochemical networks.

## Background

Recent experiments on gene and enzyme activity at single cell resolution have revealed the inherent randomness of key cellular processes linked to gene expression [1-3]. The experiments show that populations with identical genotypes present heterogeneous phenotypes and that noise at the molecular level, due to low copy numbers, contributes to population diversity. For mathematical models to capture this variability, a stochastic description is required.

Stochastic models in Computational Biology are usually based on the Master Equation (ME) of the chemical reaction kinetics [4-6]. Formally, the ME is a differential form of the Chapman-Kolmogorov equation, which gives the time evolution of *P*(**x**, *t*), the probability of the state of the system **x**. Only a handful of analytical solutions of the ME have been found and one must usually resort to approximations or numerical solutions. The most popular numerical procedure is Gillespie's Stochastic Simulation Algorithm (SSA) [7,8], a kinetic Monte Carlo algorithm that provides exact stochastic realizations of the underlying system of reactions. Each run of the SSA produces a time trace for the system; a collection of independent runs can be used to obtain convergent statistics of the time-dependent solution of the ME. In many situations, one is interested in the steady state properties of the system, i.e., in the stationary distribution of the ME, *π*. Although in principle *π *could be obtained as the first left eigenvector of the transition matrix, this computation is infeasible for most problems of interest due to the combinatorial explosion of the state space [9]. To circumvent this problem, it has become customary to sample *π *by running the SSA for a 'very long time', convincing oneself through different heuristics that stationarity has been attained. However, the SSA does not provide guarantees or information about how long the algorithm must run to converge to *π*. In recent years there has been an increased interest in finding algorithms which can address the issue of sampling from stationarity, e.g., a strategy based on forward flux sampling [10].

In a seminal paper in the field of Markov Chain Monte Carlo, Propp and Wilson introduced the idea of *Coupling From The Past *(CFTP), an ingenious procedure that provides *guaranteed *sampling from the stationary distribution of a Markov chain by running coupled chains from all possible initial conditions from the past [11]. Algorithms that guarantee sampling from the stationary distribution of a Markov chain are referred to as *Perfect Sampling *algorithms [11-14]. Recently [15], we introduced a Perfect Sampling algorithm for the SSA of biochemical networks based on Kendall's Dominated CFTP (DCFTP) [13]. This paper expands on our previous work by providing an explicit implementation of the algorithm together with a mathematical proof of its applicability to a class of reactions prevalent in models of gene regulation. We also study its numerical properties through a series of expanded examples drawn from Systems and Synthetic Biology.

## Preliminaries and definitions

### Dominated Coupling From The Past (DCFTP)

We give here a brief introduction to the CFTP framework (see [11-13] for full proofs).

The central idea behind CFTP is to find a time in the past such that the whole state space is mapped to the same state at the present, for a given sequence of random numbers. When that occurs, the state at the present can be considered to be a sample of the stationary distribution. More formally, consider a Markov process defined by the transition rule **X**_*t*+1 _= *ϕ*(**X**_*t*_), where **X**_*t *_≡ **x**(*t*) is shorthand for the state of the system at time *t*. Any Markov chain $X_{-T}^{-\infty }\equiv \{X_{-\infty },...,X_{-T}\}$, started from *t *= -∞ will have reached stationarity at time *t *= -*T*. If a chain with an unknown value **X**_-*T *_is continued to run until *t *= 0, it will attain a value **X**_0 _= *ϕ*^*T *^(**X**_-*T*_), which also comes from the stationary distribution. The CFTP algorithm searches for a time -*T *such that the composite function *ϕ*^*T *^(**X**_-*T*_) has a unique image *for all *arguments **X**_-*T*_. This implies that the chain started at -*T *is equivalent to a chain started from *t *= -∞, since it will reach the same state **X**_0 _regardless of its value at *t *= -*T*. Hence the sample **X**_0 _comes from the stationary distribution. Starting from the past and running into the present might seem counterintuitive and unnecessarily complicated. However, it is key for the algorithm to work and it can be shown that starting at *t *= 0 and coupling into the future will not guarantee that the samples are unbiased.

For large state spaces it is infeasible to monitor all initial conditions at time -*T*. However, this can be done efficiently if one can find a partial ordering over the state space that is preserved by the transition rule [12]:

$$\begin{array}{cccc} X_{t}≽Y_{t} & \Rightarrow & X_{s}≽Y_{s}, & \forall s\geq t, \end{array}$$

where ≽ denotes the partial order, i.e., a binary relation which is reflexive, anti-symmetric and transitive, although it does not necessarily satisfy total comparability. Under these conditions, the whole state space can be monitored by checking for the coalescence of coupled Markov chains started at the upper and lower extremes of the state space [11,16].

Two Markov chains are said to be coupled if they use the same sequence of random numbers and the same transition rule but are started from different initial conditions. Coupled chains that meet at a time *T*_*c *_are said to coalesce and will have identical states for *t *> *T*_*c*_. A necessary (but not sufficient) condition for the preservation of the partial ordering is that the transition function is either monotone or anti-monotone:

$$Monotonic:X_{t}≽Y_{t}\Rightarrow \phi (X_{t})≽\phi (Y_{t})$$

$$Anti\text{-}Monotonic:X_{t}≽Y_{t}\Rightarrow \phi (X_{t})≼\phi (Y_{t}),$$

for coupled chains **X **and **Y**. If the partial order is preserved, we can monitor only the paths started at the 'extremes' of the state space, since all the paths in between remain bounded by them. We therefore define *upper *and a *lower *coupled Markov chains that enclose all other paths:

$$\begin{array}{ll} \text{Lower path }(L_{t}^{-T})\text{ started from} & L_{-T}=\hat{0}\\ \text{Upper path }(U_{t}^{-T})\text{ started from} & U_{-T}=\hat{1}, \end{array}$$

where $\hat{0}≼x≼\hat{1}$, ∀**x**.

The preservation of the partial order implies two important properties for coupled chains:

*Sandwiching*: all paths started between **L **and **U **will have coalesced by the time **L **and **U **do,

(1) $$\begin{array}{cc} L_{t}^{-T}≼X_{t}^{-T}≼U_{t}^{-T}, & t>-T. \end{array}$$

*Funneling*: all paths will get closer if they are started further back into the past,

(2) $$\begin{array}{r} L_{t}^{-T}≼L_{t}^{-T-S}≼U_{t}^{-T-S}≼U_{t}^{-T},\\ t>-T,S>0. \end{array}$$

If the state space is unbounded from above, we need to use Kendall's DCFTP construction. DCFTP works by introducing a time-evolving dominating process **D **with known stationary distribution, which provides a random upper bound to the state space. The original process **X **can then be generated as an adapted functional F of the dominating process and a *mark process ***M**:

(3) $$D_{t}^{-T}≽X_{t}^{-T}=F(D_{s},M_{s}),-T\leq s\leq t.$$

The mark process generates a uniform random number each time **D **is changed. These marks are used to update the original process **X **according to the adapted functional (3) in a process that is equivalent to the direct simulation of **X **[12]. Heuristically, the DCFTP scheme works as follows. Since the dominating process is started from the stationary distribution at *t *= -*T*, $D_{t}^{-T}$ is equivalent to a process started from *t *= -∞. By the funneling property, all chains from the original process started from *t *= -∞ will be beneath the dominating process: $X_{-T}^{-\infty }≼D_{-T}^{-\infty }$. If we set **U**_-*T *_= **D**_-*T *_and **L**_-*T *_= $\hat{0}$ and check that these two extreme paths coalesce, then all chains started from *t *= -*T *map to the same state at *t *= 0, due to the sandwiching property. It then follows that $X_{0}^{-T}$ is equivalent to $X_{0}^{-\infty }$ and the sample comes from the stationary distribution of **X**, due to the equivalence of the adapted functional and the original process. Note that if **D **can be chosen to be a constant process equal to the maximal element of the state space, we obtain the CFTP algorithm [13].

These results are summarized in the following theorem for general DCFTP algorithms [12,13]:

**Theorem 1 (DCFTP) ***Consider a stationary dominating process ***D**, *for which *$\hat{0}$*is an ergodic atom, and an associated random mark process ***M**. *Suppose that the processes *L≼X≼U*are produced from ***D ***and ***M ***according to the adapted functional (3) so that the sandwiching and funneling properties (1)–(2) are fulfilled. Suppose further that ***X ***converges weakly to an invariant distribution π as t *→ ∞. *Then ***L ***and ***U ***will coalesce almost surely in finite time and, if coalescence is achieved*, **L**_0 _= **U**_0 _*is a sample from the stationary distribution π*.

**Proof **See [13].

### Stochastic Simulation Algorithm (SSA)

This section presents briefly the classic Gillespie algorithm (SSA) for the exact simulation of the Master Equation of chemical reaction networks [7].

**Definition 2 (Chemical reaction network) ***A system of chemical reactions *N*is fully specified by the tuple *N={S,R,Φ,ν}, *where *S = {*S*_1_,...,*S*_*m*_} *is a set of m different molecular species interacting through r reaction channels *R = {*R*_1_,...,*R*_*r*_}. *Each reaction R*_*i *_*is described by a stoichiometry vector ν*_*i*_, *which gives the change in the number of molecules of all species when reaction R*_*i *_*occurs, and a propensity function *Φ_*i*_(**x**), *which gives the state-dependent probability that reaction R*_*i *_*occurs. The state of the system is given by ***X**_*t *_≡ **x**(*t*) = (*x*_1_(*t*),...,*x*_*m*_(*t*)) ∈ ℕ^*m*^, *where each component x*_*i*_(*t*) *indicates the number of molecules of S*_*i *_*at time t*.

Under the assumption that the molecules are confined to a well-stirred volume and held at constant temperature, we can formulate a ME governing the evolution of the system [7]:

(4) $$\frac{dP(x,t)}{dt}=\sum_{i=1}^{r}\left[\Phi_{i}(x-\nu_{i})P(x-\nu_{i};t)-\Phi_{i}(x)P(x;t)\right]$$

The ME is a conservation equation for the probability distribution and the right hand side accounts for the rate of change of the probability of finding the system in state **x**.

A general procedure to obtain exact realizations of Markov processes first suggested by Doob [17] was applied to chemical reactions by Gillespie in his celebrated Stochastic Simulation Algorithm [7]:

**Algorithm 3 (SSA) ***Given a chemical reaction network *N={S,R,Φ,ν}, *as in Definition 2, with initial state *$X_{t_{0}}$*and stopping time T*_*s*_:

   *k *← 0

   ***loop***

      *k *← *k *+ 1

      *V*_*k*_, ${V^{\prime }}_{k}$ ~ *U*(0, 1)

      ***for ****i *= 1 *to r ****do***

         $\theta_{i}\leftarrow \sum_{j=1}^{i}\Phi_{j}(X_{t_{k-1}})$

      ***end for***

      *t*_*k *_← *t*_*k*-1 _- (1/*θ*_*r*_) log *V*_*k*_

      ***if ****t*_*k *_> *T*_*s *_***then***

         ***return ***$X_{t_{k-1}}^{t_{0}}$

      ***else***

         $X_{t_{k}}\leftarrow X_{t_{k-1}}+\nu_{i},R_{t_{k}}\leftarrow R_{i}|\frac{\theta_{i-1}}{\theta_{r}}<{V^{\prime }}_{k}<\frac{\theta_{i}}{\theta_{r}}$

      ***end if***

   ***end loop***

A run of the SSA uses the uniform random numbers *V*, *V' *to generate a random sequence of reactions **ℜ **= $\left\{R_{t_{1}},...,R_{t_{n}}\right\}$, taking place at the random transition times {*t*_1_,...,*t*_*n*_} such that *t*_*n *_<*T*_*s *_<*t*_*n*+1_. The path $X_{T_{s}}^{t_{0}}\equiv \{X_{t_{0}},X_{t_{1}},...,X_{t_{n}}\}$ is an exact stochastic realization of Eq. (4). Note that the sequence of reactions **ℜ **uniquely determines $X_{T_{s}}^{t_{0}}$. For convenience, we have committed a slight of abuse of notation when using real valued indices to denote the state $X_{t_{k}}$ and reaction $R_{t_{k}}$ taking place at time *t*_*k*_.

Henceforth, we represent compactly the SSA Markov process implemented by Algorithm 3 as:

(5) $$X_{T_{s}}^{t_{0}}=G_{\text{SSA}}(N,X_{t_{0}},T_{s}).$$

For an arbitrary initial state $X_{t_{0}}$, repeated runs of the SSA will produce convergent estimates (in the Monte Carlo sense) of the distribution *P*(**x**, *t*|$X_{t_{0}}$, *t*_0_), ∀*t *∈ [*t*_0_, *T*_*s*_] [8]. However, if one is interested in the stationary distribution *π*, running the SSA repeatedly from different initial conditions for a finite time *T*_*s *_does not guarantee that *P*(**x**, *T*_*s*_) will converge to *π*, unless the starting points $X_{t_{0}}$ are themselves drawn from *π*. Our Perfect Sampling algorithm addresses this issue.

## Proof of the DCFTP-SSA for a class of networks of biochemical reactions

Viewing the SSA as the Markov process described by (5), we have developed a specific DCFTP algorithm that provides guaranteed sampling from the stationary distribution of the corresponding chemical ME [15]. We now provide a rigorous proof and an explicit implementation of the DCFTP-SSA for an important class of biochemical reactions relevant in gene regulation.

### Partial ordering

We use the Pareto dominance relation, frequently used in economics, which is defined componentwise:

**Lemma 4 (Partial order) ***Given ***x**, **y **∈ ℕ^*m*^, *the relation *x≽y*if x*_*i *_≥ *y*_*i*_, ∀*i is a partial order*.

**Proof **The proof follows trivially from the properties of natural numbers:

Reflexivity: ∀*x*_*i *_∈ ℕ, *x*_*i *_≥ *x*_*i*_, whence x≽x

Anti-symmetry: ∀*x*_*i*_, *y*_*i *_∈ ℕ, if *x*_*i *_≥ *y*_*i *_and *y*_*i *_≥ *x*_*i *_then *x*_*i *_= *y*_*i*_. This means that x≽y and y≽x implies **x **= **y**

Transitivity: ∀*x*_*i*_, *y*_*i*_, *z*_*i *_∈ ℕ, if *x*_*i *_≥ *y*_*i *_and *y*_*i *_≥ *z*_*i *_then *x*_*i *_≥ *z*_*i*_. And the same property applies to the vectors: x≽y and y≽z implies x≽z.   □

### Assumptions on the reaction network

Consider a system of chemical reactions as given by Definition 2 with state vector **x**(*t*) ∈ ℕ^*m*^. To guarantee the preservation of the Pareto partial order under the SSA Markov process (5), we restrict ourselves to a class of chemical networks with the following properties:

(*a*) all reactions are *uni-molecular birth-death *processes with non-zero propensities, i.e., each reaction *R*_*i *_will only modify one species *S*_*j *_by adding or subtracting one molecule. The reactions can be divided into two subsets:

$$\begin{array}{lll} \text{Birth}: & & \\ R^{+} & = & \left\{R_{i}|\nu_{i}=(0,...,0,+1,0,...,0\right)\\ & \Rightarrow & x+\nu_{i}≽x\},\\ \text{Death}: & & \\ R^{-} & = & \left\{R_{i}|\nu_{i}=(0,...,0,-1,0,...,0\right)\\ & \Rightarrow & x+\nu_{i}≼x\} \end{array}$$

(*b*) the system must be *chemically reversible*, i.e., every reaction must be reversible leading to an irreducible Markov process

(*c*) all death reactions must be linear, i.e.

Φ_*i *_= *k*_*j*_*x*_*j*_   for   *R*_*i *_≡ *X*_*j *_→ ∅

(*d*) all birth reactions must have *(anti-)monotonic, sub-linear propensity functions*, i.e., ∀*i*, *j*, ∀**x**: ∂Φ_*i*_(**x**)/∂*x*_*j *_does not change sign and Φ_*i *_can be bounded by a linear function (or a constant).

As shown below, the last two assumptions are related to domination by a linear network which is required to have a stationary distribution.

Although assumptions (*a*) – (*d*) might appear restrictive, the specified class of reactions is generic and encompasses the standard equations used in the modelling of genetic and regulatory networks, the cellular circuits where stochasticity is most significant. Note that assumption (*c*) is not unrealistic for models of gene regulatory networks, in which linear death terms due to the cellular environment are prevalent. Birth reactions in these models are usually represented through *nonlinear*, uni-molecular (compound) rate laws that appear from quasi steady-state approximations. These functional forms have been shown to work well in the stochastic setting [18]. Our own simulations confirmed that they provide a good approximation in a wide range of parameters (results not shown). These compound rate laws are the key components that encode the positive and negative feedback in gene regulation. Classic examples are the sigmoid functions:

(6) $$\begin{array}{cc} \text{Monod positive feedback}: & m(x)=\frac{kx^{\alpha }}{\theta^{\alpha }+x^{\alpha }}, \end{array}$$

(7) $$\begin{array}{cc} \text{Hill negative feedback}: & h(x)=\frac{k\theta^{\alpha }}{\theta^{\alpha }+x^{\alpha }}, \end{array}$$

which are sub-linear, (anti-)monotonic functions.

### Dominating process and adapted functionals

As stated above, assumption (*d*) is related to domination. In general, the state space of chemical reaction networks is unbounded from above; hence we must use the DCFTP construction, which requires a dominating process **D **with known stationary distribution. Fortunately, it has been shown that any network of *linear *first order reactions has a stationary distribution which is multivariate Poisson [19]. Moreover, it can be shown that $\hat{0}$ is an ergodic atom for the multivariate Poisson, as assumed in Theorem 1 [13]. It then follows that a dominating process for any reaction network N={S,R,Φ,ν} composed of uni-molecular, sub-linear, (anti-)monotonic birth-death processes, as defined above, can be found by 'linearizing' the original network; that is, by constructing a linearized version of this network $\tilde{N}=\{S,R,\tilde{\Phi },\nu \}$, with the same reactions and compounds but with linear propensities ${\tilde{\Phi }}_{i}$(**x**) ≥ Φ_*i*_(**x**), ∀**x**, ∀*i *that bound the original Φ from above. Under conditions of stability, the ME of $\tilde{N}$ will have a stationary distribution $\tilde{\pi }$, given by a multivariate Poisson that can be obtained by solving a system of linear equations [19]. The existence of the stationary distribution of the dominating linear network $\tilde{N}$ guarantees the existence of the stationary distribution for the original network of reversible, uni-molecular nonlinear reactions N.

The *dominating process ***D **is defined as the stationary SSA process (5) of the linearized network $\tilde{N}$ with initial state sampled from $\tilde{\pi }$:

(8) $$\begin{array}{cc} D_{T}^{t_{0}}=G_{\text{SSA}}(\tilde{N},D_{t_{0}},T), & D_{t_{0}}\sim \tilde{\pi }, \end{array}$$

with the sequence of reactions $\tilde{ℜ}=\{{\tilde{R}}_{t_{1}},...\}$.

It has been shown [13] that a correct realization of the original (nonlinear) SSA process **X **for a network N with monotonic propensities can also be obtained through an *adapted functional *F defined in terms of the dominating process **D **and a random mark process $M=\{M_{t_{1}},...\}$ where $M_{t_{k}}$ ~ *U*(0, 1):

$$X_{T}^{t_{0}}=F(D_{T}^{t_{0}},M).$$

The update rule for F uses the ratio of the monotonic propensity functions of the original and dominating processes as follows:

(9) $$\begin{array}{l} X_{t_{k}}=\phi_{F}(X_{t_{k-1}},D_{t_{k-1}},M_{t_{k}})\\ =\{\begin{array}{ll} X_{t_{k-1}}+\nu_{t_{k}} & \text{if }M_{t_{k}}<\Psi_{t_{k}}(X_{t_{k-1}},D_{t_{k-1}})\\ X_{t_{k-1}} & \text{otherwise}, \end{array} \end{array}$$

where $\Psi_{t_{k}}(X_{t_{k-1}},D_{t_{k-1}})\equiv \Phi_{t_{k}}(X_{t_{k-1}})/{\tilde{\Phi }}_{t_{k}}(D_{t_{k-1}})$ and $\nu_{t_{k}}$, $\Phi_{t_{k}}$, ${\tilde{\Phi }}_{t_{k}}$ correspond to reaction ${\tilde{R}}_{t_{k}}$ in the reaction sequence $\tilde{ℜ}$.

The necessary ingredient for the DCFTP is the construction of an order-preserving Markov process for the evolution of two chains **X **and **Y **coupled to the dominating process **D**. For our network N, this process is defined as:

(10) $$(X_{T}^{t_{0}},Y_{T}^{t_{0}})=\hat{F}(D_{T}^{t_{0}},M),$$

with transition rule:

(11) $$\begin{array}{l} \left(X_{t_{k}},Y_{t_{k}})=\phi_{\hat{F}}(X_{t_{k-1}},Y_{t_{k-1}},D_{t_{k-1}},M_{t_{k}}\right)\\ =\{\begin{array}{l} \begin{array}{l} \left(\phi_{F}(X_{t_{k-1}},D_{t_{k-1}},M_{t_{k}}),\phi_{F}(Y_{t_{k-1}},D_{t_{k-1}},M_{t_{k}})\right)\\ \text{if }{\tilde{R}}_{t_{k}}\text{ is monotone} \end{array}\\ \begin{array}{l} \left(\phi_{F}(Y_{t_{k-1}},D_{t_{k-1}},M_{t_{k}}),\phi_{F}(X_{t_{k-1}},D_{t_{k-1}},M_{t_{k}})\right)\\ \text{if }{\tilde{R}}_{t_{k}}\text{ is anti-monotone}, \end{array} \end{array} \end{array}$$

where the componentwise transition rule is given in Eq. (9). The transition rule $\phi_{\hat{F}}$ incorporates the cross-over scheme in which the processes **X **and **Y **use the state of each other when determining their update, as introduced by Häggström and Nelander to deal with anti-monotonic processes [20].

### Proof

We now show that the partial ordering defined in Lemma 4 is indeed preserved under the evolution given by Eqs. (10)–(11) for the class of reactions specified above.

**Lemma 5 (Preservation of partial ordering) ***Consider a chemically reversible reaction network *N*of uni-molecular, sub-linear, (anti-)monotone birth-death reactions and its associated SSA dominating process ***D**, *obtained from the linearized network *$\tilde{N}$, *with the sequence of events *$\tilde{ℜ}=\{{\tilde{R}}_{t_{1}},...\}$. *Consider two coupled chains ***X ***and ***Y ***evolving under (10)–(11), where *$M=\{M_{t_{1}},...\}$*is a sequence of random marks. Then *$X_{t}≽Y_{t}\Rightarrow X_{s}≽Y_{s}$, ∀*s *> *t*.

**Proof **Assume $X_{t_{0}}≽Y_{t_{0}}$ throughout. First consider the case when ${\tilde{R}}_{t_{1}}$ is monotonic. Then the possible outcomes for *t*_0 _<*s *<*t*_1 _are:

$$\begin{array}{cc} \left(i\right) & \Psi_{t_{1}}(Y_{t_{0}},D_{t_{0}})\leq \Psi_{t_{1}}(X_{t_{0}},D_{t_{0}})\leq M_{t_{1}}\\ \left(ii\right) & \Psi_{t_{1}}(Y_{t_{0}},D_{t_{0}})\leq M_{t_{1}}<\Psi_{t_{1}}(X_{t_{0}},D_{t_{0}})\\ \left(iii\right) & M_{t_{1}}<\Psi_{t_{1}}(Y_{t_{0}},D_{t_{0}})\leq \Psi_{t_{1}}(X_{t_{0}},D_{t_{0}}). \end{array}$$

Outcome (*i*) means that neither **X **nor **Y **is modified and the preservation of the partial order is obvious. For (*iii*), both are modified by the same amount $\nu_{t_{1}}$ and the order is preserved. The interesting case is (*ii*) for which **X **is modified but not **Y**. If ${\tilde{R}}_{t_{1}}\in R^{+}$, then $X_{t_{1}}≽X_{t_{0}}≽Y_{t_{1}}=Y_{t_{0}}$ which also implies order preservation. However, if ${\tilde{R}}_{t_{1}}\in R^{-}$, then it is possible for the two chains to coalesce if $X_{t_{0}}+\nu_{t_{1}}=Y_{t_{0}}$. Note that since, by uni-molecularity, only unit changes of the states are allowed, it is impossible for two paths to cross.

When ${\tilde{R}}_{t_{1}}$ is anti-monotone, the outcomes are:

$$\begin{array}{cc} \left(iv\right) & \Psi_{t_{1}}(X_{t_{0}},D_{t_{0}})\leq \Psi_{t_{1}}(Y_{t_{0}},D_{t_{0}})\leq M_{t_{1}}\\ \left(v\right) & \Psi_{t_{1}}(X_{t_{0}},D_{t_{0}})\leq M_{t_{1}}<\Psi_{t_{1}}(Y_{t_{0}},D_{t_{0}})\\ \left(vi\right) & M_{t_{1}}<\Psi_{t_{1}}(X_{t_{0}},D_{t_{0}})\leq \Psi_{t_{1}}(Y_{t_{0}},D_{t_{0}}). \end{array}$$

As above, outcomes (*iv*) and (*vi*) lead to no change in relative order. For (*v*), again we update **X **but not **Y **due to the crossover scheme. As for the monotone case, if ${\tilde{R}}_{t_{1}}\in R^{+}$ this leads to order preservation, while if ${\tilde{R}}_{t_{1}}\in R^{-}$ it is possible for the two chains to coalesce.

It thus follows that **X **and **Y **maintain their partial ordering through every update of the (anti-)monotone process. The proof for *s *> *t*_1 _follows by induction.   □

Note that the dominated processes given by Eqs. (9) and (11) become identical when **X **= **Y**. Therefore, after coalescence the dominated process is statistically identical to the original SSA process. Since we have found a dominating process and an adapted functional, we can use Theorem 1 to obtain:

**Theorem 6 (DCFTP-SSA) ***Under the assumptions of Lemma 5, Theorem 1 is fulfilled and the DCFTP-SSA described in Algorithm 7 will produce a sample from the stationary distribution of the original process ***X ***with a coalescence time which will be finite almost surely*.

**Proof **The sandwiching (1) and funneling (2) properties follow from the preservation of partial ordering (Lemma 5) [12]. The remainder can be adapted from the general Theorem 1.   □

### Algorithm

A brief outline of the DCFTP-SSA is as follows:

**Algorithm 7 (DCFTP-SSA) ***Given a reversible system of uni-molecular birth-death chemical reactions *N*with (anti-)monotone, sub-linear propensity functions, obtain its linearized version *$\tilde{N}$*with multivariate Poisson stationary distribution *$\tilde{\pi }$:

   *T *← 1

   ${\overset{⌣}{D}}_{0}^{0}\sim \tilde{\pi },{\overset{⌣}{M}}_{0}^{0}\sim U(0,1)$

   ${\overset{⌣}{D}}_{T}^{0}\leftarrow \text{Extend}({\overset{⌣}{D}}_{0}^{0},T)$

   ${\overset{⌣}{M}}_{T}^{0}\leftarrow \text{GenerateMarks}({\overset{⌣}{M}}_{0}^{0},T)$

   ***loop***

      $\left(D_{0}^{-T},M_{0}^{-T})\leftarrow \text{Reverse}({\overset{⌣}{D}}_{T}^{0},{\overset{⌣}{M}}_{T}^{0}\right)$

      $\left(L_{0}^{-T},U_{0}^{-T})\leftarrow \text{Evolve}(D_{0}^{-T},M_{0}^{-T}\right)$

      ***if *U**_0 _= **L**_0 _***then***

         ***return *L**_0_

      ***end if***

      *T *← 2*T*

      ${\overset{⌣}{D}}_{T}^{0}\leftarrow \text{Extend}({\overset{⌣}{D}}_{T/2}^{0},T/2)$

      ${\overset{⌣}{M}}_{T}^{0}\leftarrow \text{GenerateMarks}({\overset{⌣}{M}}_{T/2}^{0},T/2)$

   ***end loop***

The function Extend(${\overset{⌣}{D}}_{T/2}^{0}$, *T*/2) runs Algorithm 3 for the linearized network $\tilde{N}$ and appends the path $G_{SSA}(\tilde{N},{\overset{⌣}{D}}_{T/2},T/2)$ to the path ${\overset{⌣}{D}}_{T/2}^{0}$. Similarly, the function GenerateMarks appends paths generated from a uniform distribution to extend the mark process. Both the marks and the forward dominating path are then reversed in time by the function Reverse. Extending these processes *backwards *in time in this manner is justified because of their stationarity and reversibility, which allows us to reverse the processes and translate them in time [9]. Finally, the Evolve function starts the coupled upper and lower chains from **L**_-*T *_= $\hat{0}$ and **U**_-*T *_= **D**_-*T *_and evolves them forward as described by Eq. (11). Note that the assumption of reversibility of the network ensures that the reverse process will be forward-evolvable. Our requirement that propensities are non-zero also ensures that reactions are not eliminated from the network. If this were to happen, it would effectively make the system irreversible. If **L **and **U **have not coalesced at *t *= 0, **D **and **M **are extended further back in time and **L **and **U **are restarted. Doubling the starting time at each iteration has been shown to be reasonably efficient (see [11] for a discussion).

## Applications of the algorithm

### Numerical convergence: First order reaction

To characterize numerically the convergence properties of the DCFTP-SSA, consider the first order reaction where species *A *is created at a (normalized) constant rate *k *from a source and degraded to a sink:

(12) $$\begin{array}{lll} & & \emptyset \overset{k}{\underset{}{\to }}A\overset{1}{\underset{}{\to }}\emptyset \\ {\dot{P}}_{j} & = & kP_{j-1}-kP_{j}+(j+1)P_{j+1}-jP_{j}\\ & \equiv & (E^{-1}-1)kP_{j}+(E-1)jP_{j}. \end{array}$$

Here *P*_*j *_denotes the probability of having *j *molecules of *A *and E and $E^{-1}$ are step operators [4]:

E*f*(*j*) = *f*(*j *+ 1) and $E^{-1}$*f*(*j*) = *f*(*j *- 1) acting on a function *f*(*j*). For the usual initial condition with 0 molecules, the time-dependent solution of Eq. (12) is a Poisson distribution with time-dependent parameter *k*(1 - *e*^-*t*^) [15]. Equation (12) is an instance of the immigration-death process which appears in different settings in the stochastic processes literature.

**Figure 1:**
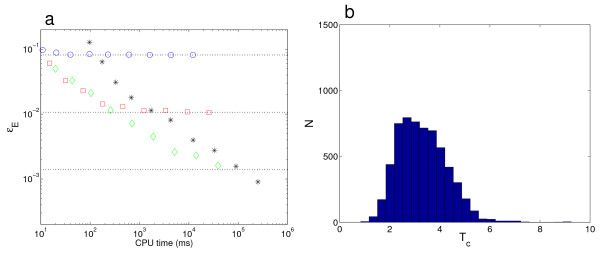
**Convergence of the DCFTP-SSA for the first order reaction (12)**. (*a*) As a function of CPU time, we represent the Euclidean error *∊*_*E *_of the stationary distribution of Eq. (12) with *k *= 5 sampled with the DCFTP-SSA (+) and with the standard SSA with stopping times *T*_*s *_= 2(○), 4(□), 6(◇). For this simple ME, the limiting value of the Euclidean error of the finite-time SSA is ${∊}_{E}{\left(T_{s}\right)}^{2}=\sum_{j=1}^{\infty }{\left(\pi_{j}-P_{j}(T_{s})\right)}^{2}=I_{0}(2k)e^{-2k}-2I_{0}(2k\sqrt{\alpha })e^{-k-\alpha }+I_{0}(2k\alpha )e^{-2\alpha }$, where *α *= 1 - exp(-*T*_*s*_) and *I*_0_(*x*) is the modified Bessel function of the first kind [15]. This means that SSA simulations that are run for a time *T*_*s *_will converge to a systematic sampling error, indicated by the dotted lines. This source of error is eliminated when using the DCFTP-SSA, which shows no flooring for *∊*_*E *_and the expected *N*^-1/2 ^scaling with the number of Monte Carlo samples [26]. The guarantees provided by the DCFTP-SSA come at a modest computational cost, which is comparable to that of long SSA runs. (*b*) The distribution of coalescence times *T*_*c *_for the DCFTP-SSA is relatively symmetric and concentrated around the mean with a rapid decay for long times. The data presented corresponds to 6000 runs. This distribution reflects the benign structure of the unimodal stationary distribution of this particular ME, which makes long coalescence times unlikely.

If, as a proxy for sampling the stationary distribution *π*, one obtains samples of *P*(*j*, *T*_*s*_|0, 0) from repeated runs of the SSA for a finite time *T*_*s*_, this will lead to a systematic error that will not disappear as the number of samples (and the CPU time) is increased. The use of the DCFTP-SSA eliminates this source of error, as shown in Fig. 1a. This figure also shows that the guaranteed convergence of the DCFTP-SSA incurs a modest additional CPU cost. The increased computational cost is twofold: increased memory requirements, since we need to store the history of the dominating process as well as the sequence of random numbers used to update the coupled chains; and longer running times, since we need to extend the process backwards for an indefinite (unbounded) amount of time. Fig. 1b presents the statistics of the coalescence times for this reaction. In this simple reaction, the distribution of stopping times is relatively symmetric and concentrated around the mean value, without the long tails that would correspond to long runs started a long time into the past. As the next example shows, the distribution of coalescence times reflects the complexity of the structure of the stationary distribution.

### Multistability: Genetic toggle switch

The mutual activation and repression of groups of genes in regulatory networks can lead to multi-stability allowing cells to attain different states [5,21]. An important and difficult problem is to find the probabilities of the different states and the expected switching times. Previously [15], we applied the DCFTP-SSA to the standard toggle switch with two Hill-repressed genes [22]. We now apply the algorithm to a more complex model of two mutually activating genes [21] with a complicated activation function which is not of the standard Monod form:

$$\begin{array}{c} \emptyset \overset{f(n_{B})}{\to }A\overset{1}{\to }\emptyset \\ ⤪\\ \emptyset \underset{f(n_{A})}{\to }B\underset{1}{\to }\emptyset \end{array}$$

(13) $$\begin{array}{r} {\dot{P}}_{n_{A},n_{B}}=(E_{A}^{-1}-1)f(n_{B})P_{n_{A},n_{B}}\\ +(E_{A}-1)n_{A}P_{n_{A},n_{B}}\\ +(E_{B}^{-1}-1)f(n_{A})P_{n_{A},n_{B}}\\ +(E_{B}-1)n_{B}P_{n_{A},n_{B}}, \end{array}$$

with the activation given by

$$f(n_{i})=\gamma +\frac{\kappa_{i}n_{i}^{4}}{\kappa_{i0}+\kappa_{i1}n_{i}+\kappa_{i2}n_{i}^{2}+\kappa_{i3}n_{i}^{3}+n_{i}^{4}},$$

where *n*_*A *_and *n*_*B *_are the number of protein molecules, *γ *is the basal production rate and *κ*_*ij *_are parameters. The functional form of the activation appears as a consequence of particular properties of this system: each transcription site can be occupied by up to four monomers and becomes activated when a tetramer is bound. However, note that *f*(*n*) is monotonic and sub-linear and therefore the DCFTP-SSA is applicable.

**Figure 2:**
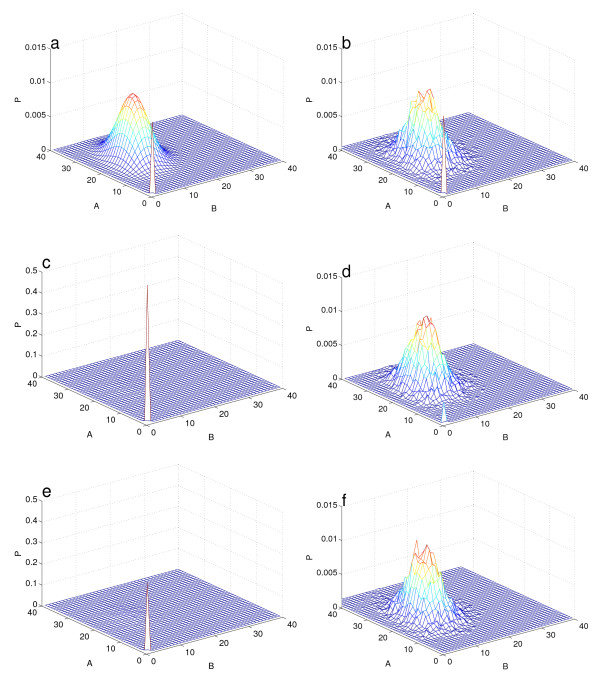
**Sampling of the stationary distribution for the bistable gene network (13) using different methods**. (*a*) The 'true' stationary probability distribution *π *for the ME (13) calculated numerically with the approximate eigenvector method [15]. The parameters are *κ*_*B *_= 25, *κ*_*A *_= 12, *κ*_*A*0 _= *κ*_*B*0 _= 60, *κ*_*A*1 _= *κ*_*B*1 _= 10, *κ*_*A*2 _= *κ*_*B*2 _= *κ*_*A*3 _= *κ*_*B*3 _= 1, and *γ *= 0.01. The locations of the two modes match the fixed points of the corresponding deterministic system. Note the extreme asymmetry of the bimodal probability distribution. (*b*) The estimate of *π *obtained from 10^4 ^samples of the DCFTP-SSA reproduces the presence of both modes and their relative weights. (*c*) Estimate of *π *from 10^4 ^samples of the SSA started at (0,0) with *T*_*s *_= 10^3^. (*d*) Estimate of *π *obtained from 10^4 ^SSA simulations started from 10^4 ^different initial conditions chosen uniformly at random on the 100 × 100 lattice closest to the origin and run for *T*_*s *_= 10^3^. (*e*) Estimate of *π *obtained from 10^4 ^SSA simulations, 5000 of them started from the origin and the other 5000 from the other mode and run for *T*_*s *_= 10^3^. (*f*) Estimate of *π *obtained from 10^4 ^samples from a long SSA run sampled at interval Δ*t *= 10^3^. Note the different scale on the *z*-axis for (*c*) and (*e*) and how the SSA runs (*c*)-(*f*) do not capture the overall structure of *π*.

**Figure 3:**
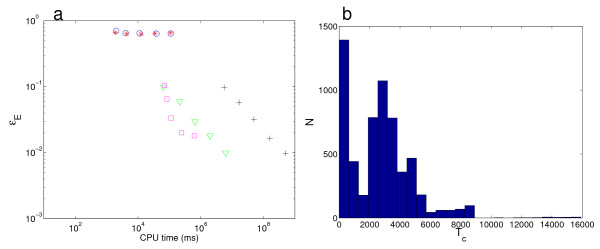
**Convergence of the DCFTP-SSA for the bistable gene network (13)**. (*a*) As a function of CPU time, we represent *∊*_*E*_, the Euclidean error of the sampled distributions estimated using: the DCFTP-SSA (+), as in Fig. 2 (*b*); the SSA with *T*_*s *_= 1000(○), as in Fig. 2 (*c*); the SSA started from the two modes (*), as in Fig. 2 (*d*); the SSA started from uniform initial conditions (∇), as in Fig. 2 (*e*); and the SSA uniformly sampled from a long run (□), as in Fig. 2 (*f*). For each scheme, we produced *N *= 100, 316, 1000, 3162 and 10000 samples to show how the error improves as the number of samples increases. The DCFTP-SSA converges to the stationary distribution at the expected *N*^-1/2 ^rate, whereas the approximate estimates obtained using the SSA level off in a similar manner as in Fig. 1*a*. (*b*) The distribution of coalescence times for the DCFTP-SSA for this network is bimodal with a very long tail for the second mode, indicating the likelihood of long coalescence times. The data presented corresponds to 6000 runs.

For certain choices of parameters, the stationary distribution of the system is bimodal: the peak located at the origin corresponds to both genes being 'off', while the other mode indicates both genes are 'on' (Fig. 2a). The extreme bimodality of this distribution makes its sampling difficult using the standard SSA. As can be seen in Fig. 3a, if we start from the initial condition (0, 0), the standard SSA levels off in a similar manner to Fig. 1a, highlighting the presence of a systematic error. In contrast, the DCFTP-SSA converges to the stationary distribution at the expected *N*^-1/2 ^rate.

Figure 2b also shows that the probability sampled with the DCFTP-SSA captures the global structure of the probability distribution even in this extreme example. On the other hand, closer inspection of the SSA simulations started from the (0, 0) reveals that for short stopping times, the process remains at the mode located near the origin (Fig 2c). Although simple heuristics on how to choose the initial condition have been suggested to improve the sampling of *π *with the SSA, Figure 2 shows that similar mis-sampling errors appear if we run the standard SSA from a variety of initial conditions. Fig. 2d shows that sampling the initial condition from a uniform grid in state space does not capture the full features of the distribution since this initial condition does not represent a consistent sampling for stationarity. If we use the fixed points of the corresponding deterministic system as initial conditions for the SSA, we would still lack the probability mass associated with each mode. For instance, starting half of the simulations at the origin and the remaining at the other fixed point provides little improvement since almost half of the simulations remain near the origin (Fig. 2e). Similar errors appear if we sample a long SSA run at fixed intervals Δ*t *to provide independent samples as intiial conditions (Fig. 2f), or even if we use samples drawn from the true stationary distribution as initial conditions for the SSA.

**Figure 4:**
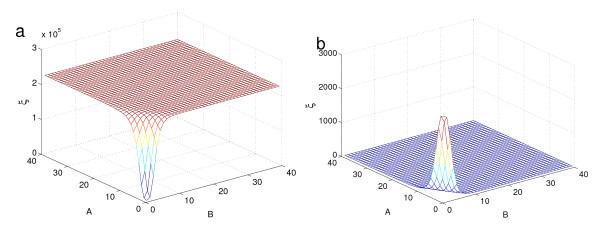
**Mean transition times for the bistable gene network (13)**. (*a*) The mean first passage time *ξ *to reach the origin for the lattice points of the state space close to the origin. The escape time from the mode located away from the origin is 2 × 10^5^. (*b*) The mean first passage time from the origin to the other mode is 3 × 10^3^.

We can understand why the stationary distribution of this system presents such a challenge for the finite-time SSA by considering the mean first passage times. Figure 4a shows the average time to reach all other states from the origin and Fig. 4b shows the average time to reach the other mode. To be certain that an SSA run will produce correct samples from the stationary distribution, it must visit each mode several times. For the system considered in Fig. 2 we need stopping times on the order of 10^7 ^to be certain that the simulation has not been stuck in one mode. With our implementation, the DCFTP compares favorably with the SSA wtih *T*_*s *_= 10^7 ^(data not shown).

Figure 3a summarizes the CPU times for the different SSA sampling schemes shown in Fig. 2 compared to the DCFTP-SSA. Again, the DCFTP-SSA introduces a reasonable overhead but provides guarantees that no systematic sampling error exists. To understand how the extreme bimodality of this distribution affects the running time of the DCFTP-SSA, Figure 3b shows the statistics of the coalescence times for this system. As compared with Fig. 1b, the distribution of coalescence times is bimodal with a second mode at long coalescence times a long tail. This reflects the complex structure of the stationary distribution in state space which induces longer coalescence times to guarantee the correct sampling. As explained in the Discussion section, the numerical performance of the algorithm in situations where long runs are more likely can be improved by the use of rejection sampling schemes.

This simple example illustrates the potential pitfalls of using the standard SSA for multimodal systems with long switching time-scales. If the SSA is run with too short stopping times, one runs the risk of missing important features of the distribution that could lead to erroneous conclusions about the number and relative weight of possible states. These problems become more acute as the dimensionality of the state space increases.

### Steady-state dynamics: Generalized repressilator

**Figure 5:**
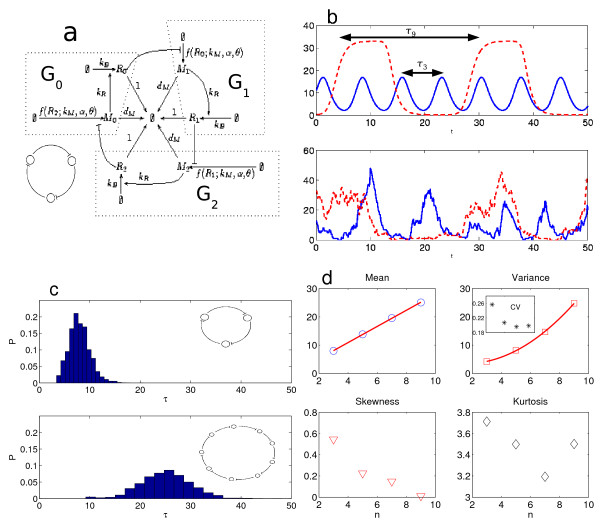
**Noise characteristics of the generalized repressilator (14)**. (*a*) Detailed diagram of the reactions in the standard repressilator with three genes involving six chemical species, as implemented with our stochastic algorithm. In the simplified cartoon, each circle represents a gene repressing the subsequent gene in a cycle. The generalized repressilator studied here considers cycles with odd number of genes *n *= 3, 5, 7, 9. (*b*) The top panel shows time series of one of the proteins for the deterministic model of the repressilator with *n *= 3 (filled) and *n *= 9 (dashed) genes with parameters *k*_*M *_= 25, *d*_*M *_= 3, *θ *= 3, *k*_*R *_= 4 and *α *= 2. The lower panel shows the corresponding time series of the SSA started from stationarity, guaranteed by the DCFTP-SSA. For the top panel, the y-axis has units of protein concentration, whereas for the lower panel the *y*-axis has unitos of number of proteins. (*c*) The top panel shows the distribution of the period for the repressilator with *n *= 3 genes, while the bottom panel shows the same distribution for the generalized repressilator with *n *= 9 genes. Note that the distribution for *n *= 3 is skewed with a long right tail, while that of *n *= 9 is more symmetric, but has fatter tails than would be expected for a Gaussian distribution. The histograms were obtained from time-series with 10^4 ^periods. (*d*) The top two panels show the dependence of the mean (∘) and variance (□) of the period distribution with *n*. The lines indicate a linear fit for the means and a quadratic fit for the variances. The inset in the top right panel, shows that, for this set of parameters, the relative noise of the period, as measured by the coefficient of variation (*), is minimal for a length of *n *= 7 genes in the loop. The two lower panels show the skewness (∇) and kurtosis (◇) for the period distribution. The skewness decreases to zero as *n *grows, in accordance with the observed decrease of the asymmetry of the distribution. The kurtosis does not disappear as *n *grows indicating the presence of long-tails. Note that the kurtosis also reaches an apparent minimum at *n *= 7.

Although regularity and robustness are important for their reliable operation in time-keeping, circadian and synchronization processes, cellular oscillators have a biochemical basis and are subject to high levels of noise [23,24]. In previous work [15], we studied the stochastic version of the repressilator, a synthetic transcriptional oscillator that consists of three mutually repressing genes in a loop (Fig. 5a) and has been implemented in *E. coli *[23]. Experiments on the original repressilator showed that the oscillations are very noisy and stochastic models are required to capture these features.

Here, we investigate the stochastic properties of the generalized repressilator with an arbitrary number *n *of genes in the loop [25]. Müller *et al *studied the deterministic version and showed that the system oscillates when *n *is odd, as expected by analogy with the ring oscillator in electronic circuits (see Fig. 5b). This system allows us to study the dependence of the variability of the oscillations with the number of genes and to showcase the scalibility of our algorithm as the number of variables (and the dimensionality of the state space) increases.

We now use the DCFTP-SSA to characterize the periodicity of the stochastic oscillations of the generalized repressilator:

(14) $$\begin{array}{lll} {\dot{P}}_{j} & = & \sum_{i=0}^{n}(E_{M_{i}}^{-1}-1)\frac{k_{M}}{\left(1+R_{i+1}^{\alpha }\right)}P_{j}\\ & + & (E_{M_{i}}-1)d_{M}M_{i}P_{j}\\ & + & (E_{R_{i}}^{-1}-1)(k_{B}+k_{R}M_{i})P_{j}\\ & + & (E_{R_{i}}-1)R_{i}P_{j}, \end{array}$$

where the shorthand *P*_*j *_denotes the state $P_{M_{0},...,M_{n},R_{0},...,R_{n}}$ and all integers are *i *mod *n*. Here *M*_*i *_are the mRNA levels (with production rate *k*_*M *_and degradation rate *d*_*M*_) and *R*_*i *_are the corresponding proteins (with basal rate *k*_*B *_and linear production rate *k*_*R*_). The repressilator network fulfills the conditions of applicability of the DCFTP-SSA and we have used our algorithm to generate time-series which are guaranteed to be at stationarity. The fact that the system has a persistent, oscillatory dynamics does not preclude it from being stationary. As expected, our DCFTP-SSA simulations show that the stationary distribution *π *conforms to the shape of a circular ridge in 2*n*-dimensional space, which is directly related to the deterministic limit cycle [4]. In this case, the probability mass is unimodal along the ridge, which means that sampling from a long time-series is unproblematic since there is no risk of avoiding regions of state space that have high probability.

As one would expect from the deterministic analysis, the mean period increases linearly with *n *(Figure 5d). This follows from the fact that the oscillatory behavior propagates in a wave-like manner around the loop. If we assume that the period is formed as the sum of *n *independent genes rising and falling in sequence, then a circuit with *n *genes will have a period whose mean scales linearly with *n*, as shown in Figure 5d in accordance with the deterministic model. However, the shape and moments of the distribution of the periods change significantly as a function of *n*, as shown in Figs. 5c–d. The distribution of the period for shorter circuits will necessarily be right-skewed since there is a minimal waiting time, akin to a refractory period, before the gene can rise again. This asymmetry is observed in the case of *n *= 3 but has almost disappeared for *n *= 9, and is captured by the skewness, which decreases towards zero as *n *increases.

Our numerics also indicate that the relative variability of the period is not constant as the number of genes in the loop increases. Figure 5d shows that the variance of the period increases quadratically, which implies that the successive periods are not independent. This implies that, for the set of parameters in Figure 5, there is an optimal length of n* = 7 genes in the loop, for which the relative fluctuations of the period, as measured by the coefficient of variation, are minimal. Note also that the kurtosis remains almost constant and positive, which indicates that there are fat tails even for longer circuits. Interestingly, the kurtosis also attains a shallow minimum at n* = 7, indicating a relative decrease in the dispersion of the distribution. Another important characteristic of an oscillator is the *rise time*, which gives an indication of its precision. Our numerics find no change in the variance of the rise times as the number of genes increases (results not shown). This is expected since the rise time of a single gene is almost independent of the preceding events unlike the period, which is an aggregated quantity and therefore more susceptible to propagated noise. The investigation of the noise characteristics of networks of transcriptional oscillators will be the object of further study.

## Discussion

The present work presents a detailed implementation of the DCFTP-SSA that could be integrated with other packages in Computational Systems Biology. We have also provided a mathematical proof of the algorithm with an explicit statement of the limits of its applicability. This detailed description is key to the extension of the algorithm to a wider class of systems. Specifically, the DCFTP-SSA can be applied to conversion reactions of the type *A *→ *B *with the realistic assumption that the monotone propensity function only depends on *n*_*A*_. Unfortunately, the extension to encompass bimolecular reactions of the type *A *+ *B *→ *C *does not seem to be trivial, since the partial ordering used in this paper will not be preserved and there is no dominating process with known stationary distribution readily available. The latter problem can be addressed partially by using the CFTP under the approximation that there is an upper bound on the number of molecules in the state space. If the bound is chosen to be large enough, it can be shown numerically that the error will be negligible. However, this approximate method will not carry the guarantees of stationarity that the DCFTP-SSA provides.

From the numerical viewpoint, the DCFTP-SSA is guaranteed to converge almost surely in finite time, but there is no upper bound on the coalescence times *T*_*c*_. Our numerics show that the distribution of coalescence times can be long-tailed when the structure of the stationary distribution is complex (Fig. 3b).

If a simulation is interrupted prematurely by an impatient user, the final sample will be biased. An alternative perfect sampling scheme is the FMMR algorithm [14], which uses rejection sampling to circumvent this problem. Our experience has shown that typically a small fraction of runs takes a very long time to converge. Being able to remove these would speed up the algorithm significantly. The bimodal example illustrates this point: if we were able to place a cut-off after the first mode, a large portion simulations would be accepted and at the same time there would be a significant save in terms of both CPU time and memory. As indicated by the examples in this paper, it is important to note that the DCFTP-SSA does not present obvious problems with scalability, as the overhead incurred to provide a certificate of stationarity is moderate. Although the computational cost of the algorithm depends on the intrinsic structure of the network, we have applied the DCFTP-SSA to various networks with several tens of variables.

In addition to producing guaranteed sampling from the stationary distribution, the DCFTP-SSA can be used to provide initial conditions for ordinary SSA runs. Since any Markov process started from stationarity will remain there for all future times, these runs are guaranteed to represent the stationary time-traces of the system. This is important for the numerical characterization of properties such as escape times and autocorrelation times of systems with high variability, e.g., with underlying multi-stable, oscillatory or excitatory behaviour [15].

## Conclusion

The SSA is an exact procedure to sample the time-dependent probability distribution of the ME of general chemical reaction networks at all times [7,8]. However, it provides no guarantees when the aim is sampling from the stationary distribution. The DCFTP-SSA presented here addresses this problem for a class of networks of relevance to genetic and enzymatic regulation. Our algorithm provides guaranteed stationary sampling and thus removes one of the sources of uncertainty in stochastic simulations. This can aid in the characterization of regulatory circuits and in the testing of model hypotheses for these systems.

## Authors' contributions

MH and MB developed the method, completed the proof and wrote the paper. MH implemented the algorithm for the simulations.
